# The Neutrophil-to-Lymphocyte Ratio and the Platelet-to-Lymphocyte Ratio as Predictors of Mortality in Older Adults Hospitalized with COVID-19 in Peru

**DOI:** 10.1155/2022/2497202

**Published:** 2022-08-03

**Authors:** Solangel Ortega-Rojas, Leslie Salazar-Talla, Anthony Romero-Cerdán, Percy Soto-Becerra, Cristian Díaz-Vélez, Diego Urrunaga-Pastor, Jorge L. Maguiña

**Affiliations:** ^1^Carrera de Medicina Humana, Facultad de Ciencias de la Salud, Universidad Científica del Sur, Lima, Peru; ^2^Grupo Estudiantil de Investigación en Salud Mental (GISAM), Sociedad Científica de Estudiantes de Medicina de la Universidad de San Martin de Porres, Universidad de San Martín de Porres, Lima, Peru; ^3^ADIECS Asociación para el Desarrollo de la Investigación Estudiantil en Ciencias de la Salud, Universidad Nacional Mayor de San Marcos, Lima, Peru; ^4^Universidad Continental, Huancayo, Peru; ^5^Facultad de Medicina, Universidad Privada Antenor Orrego, Trujillo, Peru; ^6^Dirección de Investigación en Salud, Instituto de Evaluación de Tecnologías en Salud e Investigación-IETSI, EsSalud, Lima, Peru

## Abstract

**Background:**

The prognostic value of the neutrophil-lymphocyte ratio (NLR) and platelet-lymphocyte ratio (PLR) in patients with COVID-19 is rarely described in older adults. We aimed to estimate the prognostic value of NLR and PLR, determining the mortality of adults over 60 years of age hospitalized for COVID-19 in three hospitals in Peru from March to May 2020.

**Methods:**

We performed a secondary analysis of data from a retrospective cohort carried out in Lambayeque, Peru, from March 18 to May 13, 2020. Older adults hospitalized for COVID-19 were included. The outcome variable was in-hospital mortality by all causes, while the exposure variable was the NLR and PLR (categorized in tertiles and numerically, performing a logarithmic transformation). We included sociodemographic variables, comorbidities, vital functions, laboratory markers, and treatment received during hospital stay. We evaluated the association between NLR and PLR using the hazard ratio (HR) in a Cox regression model. We estimated HR with their respective 95% confidence intervals (95% CI). We estimated cumulative/dynamic time-dependent ROC curves and reported area under the curve ROC (AUC-ROC) for 15-, 30-, and 60-day mortality with their respective simultaneous confidence intervals (confidence bands (CB)). Also, we estimated an optimal cut-off point based on the maximally selected rank statistics.

**Results:**

A total of 262 hospitalized older adults were analyzed, 71.8% (*n* = 188) of whom were male with a median age of 70 years (interquartile range: 65-78). The mean NLR and PLR were 16.8 (95% CI: 14.9-18.7; SD: 15.5) and 50.3 (95% CI: 44.6-55.9; SD: 46.3), respectively. The mortality rate was 68.7% (95% CI: 62.7-74.3). The adjusted Cox regression analysis showed that the high NLR (adjusted HR (aHR) = 2.12; 95% CI: 1.43-3.14) and PLR (aHR = 1.90; 95% CI: 1.30-2.79) tertiles were associated with a higher risk of mortality. The maximum AUC-ROC values at 60 days of follow-up for NLR and PLR were 0.713 (95%CB: 0.627-0.800) and 0.697 (95%CB: 0.583-0.754), respectively.

**Conclusions:**

The NLR and PLR are predictors of higher risk of mortality, and these results suggest that both could be reliable and practical markers for the identification of older adults at high risk of mortality by COVID-19. NLR and PLR have prognostic value, with an AUC greater than 0.5; however, by themselves, they are weak prognostic markers. It is important to carry out future studies incorporating these two markers into preexisting models or designing new ones considering them.

## 1. Introduction

Since December 2019, the number of cases diagnosed with COVID-19 worldwide has been rising, reaching 169 million confirmed cases and 3.5 million deaths [1]. On April 14, 2022, the World Health Organization (WHO) issued the latest epidemiological update for COVID-19, indicating that 48% of deaths were from Latin American and the Caribbean (LAC), which presented the highest proportion of deaths and confirmed cases with 44% and 30%, respectively [2]. In the report on the impact of COVID-19 in LAC issued by the United Nations (UN) in July 2020, the health impact in this region is described as being due to a health system that was unequal, fragmented, and insufficient to manage the health and human crisis emerging at that time [3]. Indeed, decisions regarding the use of scarce resources such as ventilators have been challenging in these countries, requiring hospitals and medical centers to consider the age of the patient, comorbidities, and the impact on specific outcomes, such as life expectancy or likelihood of survival [4].

Various risk factors, such as the presence of diabetes, hypertension, cardiovascular diseases, and organ failure, have been described in relation to clinical worsening of patients with COVID-19. In addition, male sex and advanced age have been described as nonmodifiable risk factors associated with mortality [5]. Likewise, several biomarkers have also been associated with the risk of mortality by COVID-19, including the neutrophil count, ultrasensitive C-reactive protein, erythrocyte sedimentation rate, creatinine, S-adenosylmethionine [6], lactate-dehydrogenase (LDH), creatinine-kinase, nitrate urea, D-dimer, the lymphocyte-monocyte index or ratio (LMR), the neutrophil-to-lymphocyte ratio (NLR), and the platelet-to-lymphocyte ratio (PLR) [7, 8]. The NLR and the PLR are used as biomarkers in patients with sepsis, multiorgan damage, pneumonia, cancer, cardiovascular diseases, and pregnancy complications [9, 10].

The prognostic value of the NLR and the PLR in patients with COVID-19 has been described in various articles related to severity, disease progression, and mortality, with the NLR being an effective predictor in the most severe patients and the PLR indicating the severity and prognosis of the disease during hospitalization [5, 8, 11]. It should be noted that the geriatric population has a higher risk of mortality due to the greater number of comorbidities presented. However, few studies have been conducted in this age group [12]. Several systematic reviews with meta-analyses [13–15] have described the association between the NLR and the severity and mortality of patients with COVID-19. However, these reviews did not include a subanalysis by population group and studies of LAC populations were not included. In addition, to our knowledge, no systematic reviews and meta-analysis have evaluated the association between PLR and the severity and mortality due to COVID-19. Therefore, the objective of the study was to estimate the prognostic value of the NLR and the PLR in determining the mortality of adults over 60 years of age hospitalized for COVID-19 in three hospitals in Peru from March to May 2020.

## 2. Methods

### 2.1. Study Design, Population, and Sample

We carried out a secondary data analysis of a retrospective cohort [16] performed in Peru from March 18 to May 13, 2020. The study population consisted of 263 older adults (60 years and older) hospitalized with a diagnosis of COVID-19 in the *Hospital Almanzor Aguinaga Asenjo* (third level), *Hospital Luis Heysen Incháustegui* (second level), and the *Hospital Clínica EsSalud Chepén* (second level), in Lambayeque, Peru, during the study period.

This secondary data analysis included patients with a diagnosis of COVID-19 confirmed by rapid lateral flow test or molecular testing (reverse transcription polymerase chain reaction (RT-PCR)). Likewise, we included hospitalized patients over 60 years of age with a suspected diagnosis based on a clinical or radiological pattern profile plus an epidemiological link (having had contact with a confirmed case during the last 14 days) despite having a nonreactive rapid lateral flow test for COVID-19 or a negative RT-PCR. All included patients were admitted to the hospital solely for treatment of COVID-19. Older adults who did not have the variables of interest (NLR, PLR, and confounders) were excluded. We defined zero time as the hospitalization admission, and we considered an administrative censoring time at 60 days.

### 2.2. Variables

#### 2.2.1. Outcome Variable: Mortality

We evaluated in-hospital mortality by all causes of patients hospitalized with a confirmed or probable diagnosis of COVID-19 as an outcome variable. We do not consider mortality after hospital discharge. These data were obtained from the review of the virtual medical records of patients hospitalized from March 18 to May 13, 2020.

#### 2.2.2. Exposure Variables: NLR and PLR

The NLR was calculated by dividing the neutrophil and lymphocyte counts, and the PLR was calculated by dividing the platelet and lymphocyte counts. For the present study, tertiles were used for these variables, due to the lack of a validated cut-off point for this population in the literature. We considered the first measure of laboratory markers during the first 24 hours of hospital admission.

### 2.3. Other Variables

#### 2.3.1. Sociodemographic Characteristics

We collected the following sociodemographic characteristics: age (60 to 70, 71 to 80, and 81 or more), sex (men and women), and comorbidities (type 2 diabetes mellitus, obesity, asthma, high blood pressure, chronic kidney disease, and cancer).

#### 2.3.2. Symptoms and Epidemiological Link

We collected the symptoms registered in the virtual medical records (cough, fever, shortness of breath, sore throat, headache, diarrhea, anosmia, ageusia, and nasal congestion) and the time of illness of the patients from the onset of symptoms (in days). Contact with a confirmed case of COVID-19 (no and yes) was also taken into account.

### 2.4. Baseline Vital Functions

We collected the baseline values of vital functions at patient admission registered in the virtual medical records, such as respiratory rate (tachypnea ≥ 22 and ≥30), temperature (fever ≥ 38°C), oxygen saturation (<96%, <94%, <92%, <90%, <85%, and<80%), and heart rate (tachycardia ≥ 100 and ≥120).

### 2.5. Baseline Laboratory Markers

The following laboratory markers measured in the first 24 hours of hospital admission were collected: leukocyte count (leukocytosis ≥ 10,000 cells/mm^3^), hemoglobin (g/dL), neutrophils (cells/mm^3^), platelets (thrombocytopenia < 150,000 cells/mm^3^), lymphocytes (lymphopenia < 8 cells/mm^3^), creatinine (mg/dL), LDH (≥245 and ≥450 U/L), urea (mg/dL), alanine aminotransferase (U/L), and aspartate transaminase (U/L).

### 2.6. Treatment Received

We included the treatment administered, including antibiotic therapy (cephalosporins, carbapenems, azithromycin, among others), antiparasitics (ivermectin and hydroxychloroquine), corticosteroid therapy (prednisone, dexamethasone, methylprednisolone, and hydrocortisone), antivirals (lopinavir/ritonavir), and anticoagulants (enoxaparin). In addition, we included patients admitted to the intensive care unit (ICU), those requiring high-flow oxygen (FiO_2_ ≥ 0.36), and those who required ICU (FiO_2_ ≥ 0.80).

### 2.7. Statistical Analysis

A database in Excel 2010 format was created and imported to the statistical package STATA v17.0 (StataCorp, TX). Qualitative variables were described using absolute and relative frequencies, while numerical variables were presented as means and standard deviation (SD) or medians with interquartile ranges (IQR), as appropriate.

The bivariable associations between NLR and PLR with categorical covariates were reported as mean/median comparison, as appropriate. For clinical interpretability, the NLR and PLR values were also categorized into tertiles and compared against the other covariates. The comparison of means/medians between two groups was evaluated using the Student *t*- or Mann–Whitney *U* test, as appropriate. On the other hand, comparison between proportions of qualitative variables was performed using the Pearson chi-square or the Fisher exact test, depending on the expected values.

We estimated crude and adjusted association between NLR and PLR using hazard ratios (HR) obtained from multivariable Cox regression models. Ties adjustment was done with the Efron method. The incorporation of the NLR and PLR in the model, as well as that of other numerical variables (age and oxygen saturation), was carried out in two ways. The first approach involved incorporating them as continuous variable allowing nonlinear form to avoid drawbacks of categorization. We used a multivariable fractional polynomial approach to select an appropriate power transformation for age, oxygen saturation, PLR, and NLR according Royston and Sauerbrei's methodology [17]. A closed test procedure was used to ensure that the overall type 1 error is close to nominal significance level of 5% [17]. We applied a natural logarithmic transformation of PLR and NLR to reduce high leverage values and stabilize the estimations. To show clinically interpretable results, we describe the relationship between NLR and PLR using effect plots of the adjusted hazard ratios along the observed values of these markers. The second approach to incorporate NLR and PLR in the models was chosen to get more clinically interpretable estimates. We modeled the association using the tertiles of NLR and PLR as a predictor in a multivariable cox regression. Age and oxygen saturation were introduced as nonlinear terms using the same methodology described above. Kaplan-Meier curves of overall a survival against NLR or PLR tertiles were reported. Log-rank test was used to compare these survival curves.

In total, four crude and adjusted models were developed using an epidemiological approach for each exposure variable including well-known predictors or confounders which has been described in the literature. Likelihood profile confidence intervals at 95% level and *P* values estimated via likelihood ratio tests were reported considering a 5% of significance level. The hazard proportionality assumption was evaluated by inspection of scaled Schoenfeld residuals and supplemented with the hazard proportionality hypothesis test. Plots of martingale residuals against numeric predictors and index plots of DFbetas were used to assess linearity and to detect influential points, respectively.

To explore the prognostic value of NLR and PLR to predict mortality, we estimated cumulative/dynamic time-dependent ROC curves [18] using inverse probability of censoring weighting and Kaplan-Meier estimator as implemented in package “timeROC” in R. We also reported area under the curve ROC (AUC-ROC) for 15-, 30-, and 60-day mortality with their respective simultaneous confidence intervals (confidence bands (CB)). Also, we estimated an optimal cut-off point based on the maximally selected rank statistics [19] as implemented by the “survminer” package in R. Kaplan-Meier survival curves against NLR and PLR categorized according to these optimal cut-off points also were reported.

### 2.8. Ethical Aspects

This secondary analysis was approved by the Research Ethics Committee of the *Red Prestacional Lambayeque* (CIEI-RPL: 068-DIC-2021). Since this study involved analysis of secondary data, no additional measurement was performed in the participants. In addition, the primary study was evaluated and approved by the Research Ethics Committee for COVID-19 of EsSalud, Peru (No. 42-IETSI-ESSALUD-2020).

## 3. Results

### 3.1. Descriptive and Bivariate Analysis according to Mortality in the Study Sample

**Figure 1:**
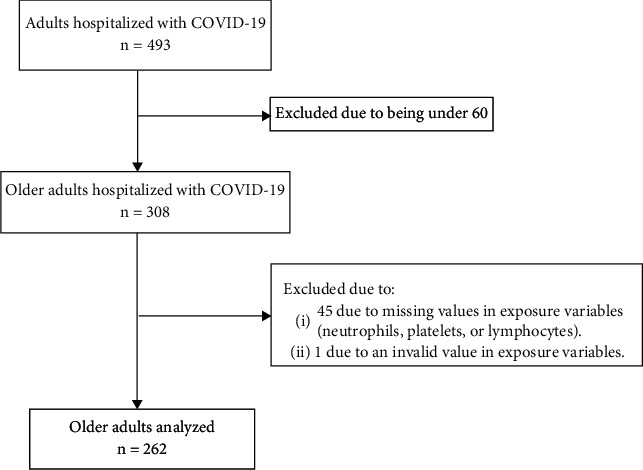
Flowchart for sample selection.

We found 308 older adults in the database; however, we excluded 46 due to missing values in exposure variables and finally analyzed a total of 262 hospitalized older adults (Figure 1). We found that 71.8% (*n* = 188) were male and the median age was 70 years (IQR: 65-78) and ranged between 60 and 98 years; 53.1% (*n* = 139) were between 60 and 70 years old, and 53.1% (*n* = 139) had no comorbidities. The most frequent comorbidity was high blood pressure in 31.3% (*n* = 82). Symptoms were presented with a median of 7 (IQR: 4-10) days before hospital admission and ranged between 1 and 30 days, the most common being respiratory distress and cough with 91.1% (*n* = 224) and 80.1% (*n* = 197), respectively. Likewise, 81.0% (*n* = 179) presented tachypnea on admission to hospital, while the median oxygen saturation was 86 (IQR: 75-90), and 33.5% (*n* = 84) presented oxygen saturation less than 80%. Confirmation of COVID-19 was obtained in 83.6% (*n* = 219) of the cases with 96.8% (*n* = 212) being diagnosed by a positive lateral flow test. Leukocytosis was presented by 63.7% (*n* = 167), lymphopenia by 45.8% (*n* = 120), and thrombocytopenia by 13.4% (*n* = 35). On the other hand, 72.9% (*n* = 191) required ICU admission but only 2.7% (*n* = 7) were admitted.

The mean NLR was 16.8 (95% CI: 14.9-18.7; SD: 15.5) and ranged between 1.9 and 49, while mean PLR was 50.3 (95% CI: 44.6-55.9; SD: 46.3) and ranged between 1.6 and 195.5. Distribution of NLR and PLR was markedly skewed to the right (Figure S1). We found that neutrophilia was 30.3% (*n* = 20), 78.4% (*n* = 69), and 90.7% (n = 98) in the low, intermediate, and high tertile of NLR, respectively. Likewise, lymphopenia was 15.2% (*n* = 10), 31.8% (*n* = 28), and 75.9% (*n* = 82) in the low, intermediate, and high tertile of PLR, respectively. On the other hand, thrombocytopenia was 30.7% (*n* = 27), 8.1% (*n* = 7), and 1.2% (*n* = 1) in the low, intermediate, and high tertile of PLR. Furthermore, lymphopenia was 21.6% (*n* = 19), 44.8% (*n* = 39), and 71.3% (*n* = 62) in the low, intermediate, and high tertile of PLR.

The 60-day cumulative-incidence mortality was 68.7% (95% CI: 62.7-74.3) (*n* = 180) in the study sample. The bivariable analysis of the study variables and mortality is shown in Table 1. Figure S2 shows the distribution of NLR and PLR according death status at the end of follow-up. Overall, distributions of levels of NLR and PLR were higher in dead comparison to alive patients.

### 3.2. Treatment Received by the Study Participants

We found that 98.9% (*n* = 259) of older adults received antibiotic therapy, with 79.1% (*n* = 205) receiving the combination of azithromycin and cephalosporins. It should be noted that 69.1% (*n* = 181) received corticosteroid therapy, while only 30.9% (*n* = 56) received dexamethasone. Hydroxychloroquine was administered to 73.3% (*n* = 192) of hospitalized older adults and 27.1% (*n* = 71) received ivermectin. Bivariate analysis showed statistically significant differences between the types of corticosteroids received, having received ivermectin or enoxaparin, and mortality (Table 2).

### 3.3. Survival according NLR and PLR Tertiles

**Figure 2:**
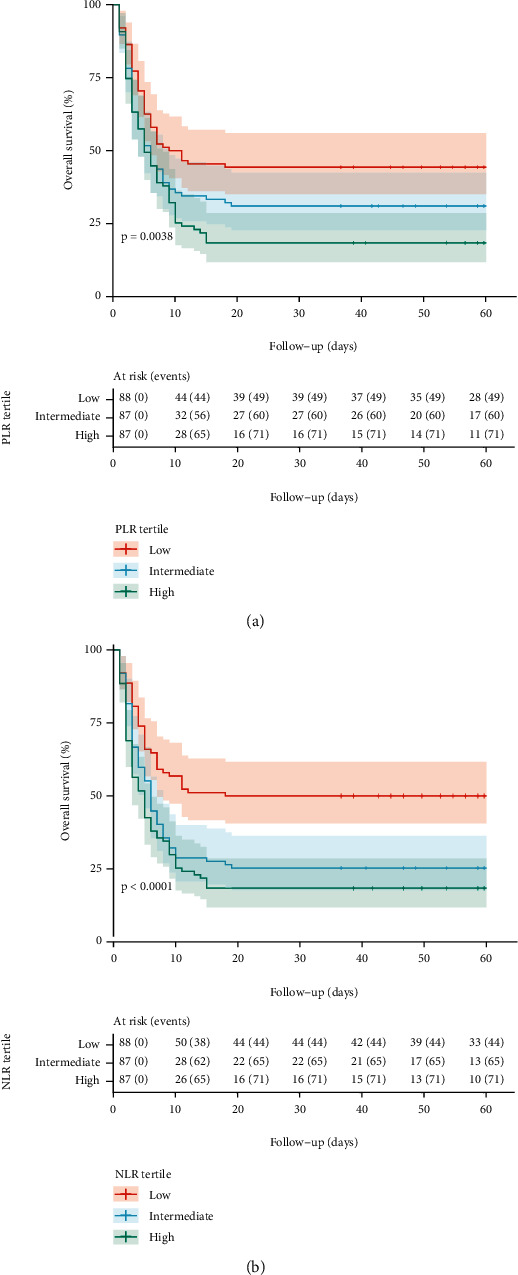
Overall survival curves by (a) platelet-to-lymphocyte ratio (PLR) tertiles and (b) neutrophil-to-lymphocyte ratio (NLR) tertiles. Overall survival curves were estimated by Kaplan-Meier method and *P* values, by log-rank test.

Participants admitted to hospital with lower NLR and PLR tertile levels presented a significantly higher overall survival compared to higher NLR (log-rank test *P* = 0.004) and PLR tertile levels (log-rank test *P* < 0.001) (Figure 2).

### 3.4. Biomarkers as Predictors for Mortality in Hospitalized Older Adults

In the crude Cox regression analysis, higher levels of NLR (crude HR (cHR) = 1.65; 95% CI: 1.37-1.98) and PLR (cHR = 1.37; 95% CI: 1.15-1.53) were associated with higher mortality risk. After controlling for sex, age, oxygen saturation, comorbidities, and treatment with dexamethasone, the adjusted hazard ratios (aHR) remain similar and significantly associated with mortality for NLR (aHR = 1.56; 95% CI: 1.30-1.88) and PLR (aHR = 1.42; 95% CI: 1.19-1.71) (Table 3). Although the increase of mortality risks associated to NLR and PLR were monotonically consistent along all these values, it was also not linear. Figure S3 shows that mortality hazard rate rapidly increases at lower values of NLR and PLR until a certain threshold is achieved in which the hazard rates remain raising more slowly. Crude functional form of the relationship between NLR and PLR against mortality was similar than adjusted estimates (Figure S4). The estimated functional forms of age and oxygen saturation in the adjusted models are shown in Figures S5 and S6.

Concordantly, with the analysis of NLR and PLR as continuous variable, we found that the intermediate (cHR = 1.89; 95% CI: 1.29-2.78) and high tertiles of the NLR (cHR = 2.37; 95% CI: 1.62- 3.45) were associated with a higher hazard rate of mortality. Likewise, the intermediate (cHR = 1.47; 95% CI: 1.01-2.14) and high tertiles of PLR (cHR = 1.84; 95% CI: 1.28-2.65) were also associated with a higher mortality risk in older adults hospitalized for COVID-19. After controlling for the same set of predictors, higher NLR (*P* = 0.017) and PLR levels (*P* = 0.004) remained significantly associated with higher mortality risk. Specifically, high NLR tertile had higher mortality risk than low tertile (aHR = 2.12; 95% CI: 1.43-3.14); and high PLR tertile had higher mortality risk than low tertile (aHR = 1.90; 95% CI: 1.30-2.79) in older adults hospitalized for COVID-19.

### 3.5. Prognostic Value of NLR and PLR to Predict 15-, 30-, and 60-Day Mortality

The AUC-ROC to predict mortality at time 15, 30, and 60 for NLR and PLR are shown in Figure S7. All AUC-ROC for NLR and PLR were significantly different of 0.5, reflecting some degree of prognostic value to predict mortality. The maximum AUC-ROC values at 60 days of follow-up for NLR and PLR were 0.713 (95%CB: 0.627-0.800) and 0.697 (95%CB: 0.583-0.754), respectively. In general, PLR had AUC-ROC values lower than NLR, but these differences were not statistically significant. The optimal cut-off point for NLR according to Youden's index was 8.6. At this value, sensitivity and specificity to predict mortality until day 60 were 79.4% (95% CI: 73.5%-85.4%) and 55.4% (95% CI: 42.3%-68.4%), respectively (Table 4). Using the maximally selected rank statistics criterion, the optimal cut-off was achieved at 6.23 (Figure S8) with an estimated sensitivity of 90.6% (95% CI: 86.3%-94.85) and a specificity of 39.3% (26.5%-52.1%). Regarding PLR, Youden's index and maximally selected rank statistics criteria coincide in the optimal cut-off point of 34.2 (Figure S9) with an estimated sensitivity and specificity of 62.8% (95% CI 55.7%-69.9%) and 69.6% (95% CI: 57.6%-81.7%). In Figure S10, we showed that participants with a higher NLR and PLR categorized according to maximal selected rank statistics had a lower survival.

## 4. Discussion

### 4.1. Main Findings

In this study of 262 older adults hospitalized in three hospitals in Peru, we found that having a high NLR or PLR value was associated with a higher risk of mortality in older adults. This is one of the first studies to evaluate the prognostic role of NLR or PLR as biomarkers of mortality in older adults with COVID-19. Our results suggest that NLR and PLR could help in the identification of older adults at higher risk of mortality. Further studies which assess the added prognostic increment value of NLR and PLR into known preexisting models, such as 4C [20] or others [21], are necessary.

### 4.2. Comparison with Previous Studies

In the present study, we found that the NLR and the PLR were associated with a higher risk of mortality in older adults hospitalized for COVID-19. In a previous study conducted in 226 older Chinese adults, the NLR was reported to be a risk factor for severe or critical illness [12]. However, these authors did not assess all-cause mortality as an outcome and did not include the PLR, which has been described in the literature as a relevant biomarker for severity and mortality in COVID-19 disease [22]. On the other hand, the role of the NLR and the PLR as biomarkers of the risk of severity and mortality by COVID-19 has been described in previous systematic reviews and meta-analyses [9, 14, 15, 23]. However, these review articles did not perform a subgroup analysis to assess the estimated risk of mortality for this important age group. Previous publications have studied the role of the NLR and the PLR in mortality in adults in Europe [24], the United States [25], Latin America [26], and even Peru [16]. However, while the study samples included older adults, they did not report an association measure exclusively for older adults, nor did they focus on this population group.

Peru was considered the world epicenter of the pandemic during the first wave [27, 28]; then, we could expect a high mortality incidence. Globally and also in Peru, the highest COVID-19 mortality was reported in older adults [29] because age is considered a risk factor for mortality in this disease [30]. In addition, Lambayeque was one of the regions with the highest seroprevalence [31]; then, we would expect higher mortality in older adults. A previous study found in-hospital mortality of 49.5% in Peruvian adults during the first wave; however, this study did not include exclusively older adults; therefore, it is totally plausible that mortality could be higher because we included only this group [32].

### 4.3. Interpretation of Results

The association between high values of both biomarkers and mortality might be explained by the role of inflammation. Inflammation has been described as part of the pathophysiology of COVID-19 and a key point to assess the prognosis of patients. Indeed, on the development of a respiratory infection, the immune system produces inflammatory markers such as granulocyte colony stimulating factor, tumor necrosis factor-alpha, interferon gamma, interleukin- (IL-) 6, and IL-8 by endothelial cells and lymphoid cells activating neutrophils and promoting their proliferation, thus, the hypothesis of the role of neutrophilia and lymphopenia in the death of patients with COVID-19 [33]. On the other hand, the increase in anti-inflammatory cytokines leads to lymphocyte apoptosis, inducing lymphocytopenia [34–37]. Therefore, the NLR is elevated as a result of an increased neutrophil count and decreased lymphocyte count [33, 36, 38, 39] as we found in the tertiles described in our results. Likewise, lymphocytopenia is more frequently described as a finding in SARS-CoV-2 infection and could lead to an elevated PLR, compared to thrombocytopenia, as described in our PLR tertile findings. It is widely recognized that thrombocytopenia and lymphocytopenia are associated with an increased risk of adverse outcomes in patients with COVID-19 [40, 41]. Platelets play an important role in the inflammatory response of damaged epithelium and can be activated in response to proinflammatory cytokines [42, 43]. Also, the interaction between proinflammatory cytokines from platelets and circulating leukocytes leads to an increased release of cytokines [44]. Finally, SARS-CoV-2 viral infection could lead to pulmonary endothelial cell injury, then activating platelet aggregation in the lungs, producing the appearance of thrombus [41, 45], and an adverse outcome in COVID-19 patients.

The NLR has also been used as a marker of prognostic value for other pathologies such as cardiovascular diseases [46] and to determine the severity of community-acquired pneumonia [47] as well as in neoplastic diseases such as colorectal cancer [48]. On the other hand, the PLR has also been considered as a biomarker in other viral diseases such as dengue or hepatitis B. The diagnosis of these diseases has been found to be correlated with low PLR values [49]. In addition, a study conducted in China in 2018 analyzed the association between PLR, among other markers, and influenza A infection. The authors found that a low PLR was associated with this viral disease and was described as being useful for its diagnosis [50].

### 4.4. Clinical Relevance of the Findings

Older adults are more susceptible to developing the most serious and critical condition of this disease leading to high mortality rates [51, 52], requiring biomarkers that can be used individually or jointly for early diagnosis and mortality prognosis. The NLR and the PLR can be calculated quickly based on the routine blood count on admission, allowing physicians to identify high-risk COVID-19 patients early, ensuring adequate clinical follow-up, and thereby reducing in-hospital mortality, especially in resource-limited settings [36, 53].

We found that the NLR and PLR have prognostic value, with an AUC greater than 0.5; however, by themselves, they are weak prognostic markers. For this reason, it is important to carry out future studies incorporating these two markers into preexisting models or designing new ones considering them. Likewise, NLR and PLR could be accessible, finding only 15% of missing values in our study sample. A previous study conducted in 210 Chinese older adults found a higher AUC (0.774) with a similar cut-off point (6.48) as a prognostic factor for severity [54]. On the other hand, various studies have suggested different NLR cut-off points (ranging from 2.97 to 4.80) for COVID-19 severity; however, these studies were not conducted in older adults [14].

It is well-known that both NLR and PLR are used as prognostic markers in different diseases. Thus, an NLR value greater than 5 has been described for colorectal cancer [55], 4.7 for melanoma [56], and 3.9 for preeclampsia [57]. On the other hand, a PLR value greater than 150 for colorectal cancer [55] and 109 for preeclampsia [57] has been described as a prognostic marker. It is also even expected to find variability between populations. All these results highlight the great variability between diseases that have these markers and justify the need to assess the cut-off point of these markers.

### 4.5. Limitations and Strengths

This study has limitations: (1) the study population corresponded only to older adults insured by EsSalud, who have particular sociodemographic characteristics, and does not represent all older Peruvian adults; however, EsSalud insures nearly a third of the Peruvian population [58]; (2) some of the variables included have missing values (as laboratory markers including neutrophils, lymphocytes, and platelets) because they were not measured at hospital admission; however, the adjusted regression model was developed considering only those with a percentage of missing values less than 20%; (3) we conducted this study with data collected during the first wave of COVID-19 in Peru, which could be relevant within the Peruvian context, in which not many articles have been published and these studies could help us better understand what happened during that pandemic period and be prepared for future pandemics; (4) this study was carried out during a prevaccine era; however, studies developed during the postvaccine distribution era described results similar to ours [59–61]; (5) among laboratory markers, some, which are considered relevant, such as cytokines, were not measured in this study; however, the variables most accessible and practical according to the literature were used. Despite these limitations, this study represents one of the first to evaluate the role of the NLR and the PLR as predictors of mortality in older adults with COVID-19 [12].

## 5. Conclusions

The NLR and the PLR are quick and practical markers for the identification of groups of older adults at high risk of mortality by COVID-19. The use of these biomarkers alone or into preexisting known models could improve the identification of who might benefit from earlier or more aggressive interventions (i.e., ICU admission). It is important to carry out future studies incorporating these two markers into preexisting models or designing new ones considering them. It should be noted that our findings correspond to patients evaluated and treated during the first wave of the COVID-19 pandemic in Peru. It is necessary to implement more timely care for older adults, as they are the most vulnerable group.

## Figures and Tables

**Table 1:** Descriptive and bivariate analysis according to vital status in a sample of older adults hospitalized for COVID-19 (*n* = 262).

Variables	*n*	%	Mean ± SD^1^	In-hospital death		*P* value
			Median (p25-p75)	Survivor	Nonsurvivor	
				*n* = 82	*n* = 180	
				31.30%	68.70%	
NLR			12.7 (8.0-19)	8.6 (4.6-14.8)	14.8 (9.6-23.4)	<0.001
Low tertile	88	33.6	6.0 (3.8-8.0)	44 (50.0)	44 (50.0)	<0.001
Intermediate tertile	87	33.2	12.7 (11.0-14.8)	22 (25.3)	65 (74.7)	
High tertile	87	33.2	23.8 (19.0-32.3)	16 (18.4)	71 (81.6)	
PLR			37.7 (20.7-61.6)	28.2 (16.5-47.2)	43.7 (25.5-67.3)	<0.001
Low tertile	88	33.6	16.3 (9.5-20.8)	39 (44.3)	49 (55.7)	<0.001
Intermediate tertile	87	33.2	38.3 (31.3-47.2)	27 (31.0)	60 (69.0)	
High tertile	87	33.2	76.3 (61.6-118)	16 (18.4)	71 (81.6)	
Sociodemographic characteristics						
Age			70 (65-78)	67 (64-72)	72 (66-80)	<0.001
≤70 years	139	53.1		57 (41.0)	82 (59.0)	<0.001
71-80 years	74	28.2		19 (25.7)	55 (74.3)	
≥81 years	49	18.7		6 (12.2)	43 (87.8)	
Sex						0.962
Female	74	28.2		23 (31.1)	51 (68.9)	
Male	188	71.8		59 (31.4)	129 (68.6)	
Comorbidities						0.111
0	139	53.1		48 (34.5)	91 (65.5)	
1	71	27.1		24 (33.8)	47 (66.2)	
2 or more	52	19.8		10 (19.2)	42 (80.8)	
Obesity	40	15.3		11 (27.5)	29 (72.5)	0.574
Type 2 diabetes mellitus	46	17.6		10 (21.7)	36 (78.3)	0.124
High blood pressure	82	31.3		24 (29.3)	58 (70.7)	0.633
Asthma	5	1.9		0 (0)	5 (100)	0.329
Cancer	6	2.3		1 (16.7)	5 (83.3)	0.669
Chronic kidney disease	7	2.7		1 (14.3)	6 (85.7)	0.44
Symptoms and epidemiological link						
Time of illness (*n* = 221)			7 (4-10)	7 (4-10)	7 (5-10)	0.915
Symptoms (*n* = 246)						
Shortness of breath	224	91.1		73 (32.6)	151 (67.4)	0.61
Cough	197	80.1		62 (31.5)	135 (68.5)	0.666
Fever	130	52.9		37 (28.5)	93 (71.5)	0.194
Sore throat	23	9.4		6 (26.1)	17 (73.9)	0.516
Diarrhea	20	8.1		7 (35.0)	13 (65.0)	0.773
Headache	13	5.3		5 (38.5)	8 (61.5)	0.761
Nasal congestion	3	1.2		0 (0)	3 (100)	0.553
Anosmia	1	0.4		0 (0)	1 (100)	1,000
Ageusia	1	0.4		0 (0)	1 (100)	1,000
Contact with a confirmed case of COVID-19						
Yes	8	3.1		3 (37.5)	5 (62.5)	0.708
Baseline vital functions						
Temperature (°C) (*n* = 153)			36.8 (36.5-37.0)	36.7 (36.5-37.0)	36.8 (36.5-37.0)	0.544
Fever (≥38°C)	14	9.2		7 (50.0)	7 (50.0)	0.25
Respiratory rate (*n* = 221)			26 (22-30)	24 (20-28)	28 (24-32)	<0.001
Tachypnea (≥22 RPM)	179	81		51 (28.5)	128 (71.5)	0.003
Tachypnea (≥30 RPM)	70	31.7		13 (18.6)	57 (81.4)	0.002
Heart rate (*n* = 248)			90 (80-106)	86 (79-94)	94 (83-110)	<0.001
Tachycardia (≥100 LPM)	82	33.1		11 (13.4)	71 (86.6)	<0.001
Tachycardia (≥120 LPM)	23	9.3		4 (17.4)	19 (82.6)	0.127
Oxygen saturation (%) (*n* = 251)			86 (75-90)	90 (87-93)	80 (70-88)	<0.001
<96%	236	94		73 (30.9)	163 (69.1)	0.205
<94%	226	90		65 (28.8)	161 (71.2)	0.001
<92%	203	80.9		52 (25.6)	151 (74.4)	<0.001
<90%	174	69.3		36 (20.7)	138 (79.3)	<0.001
<85%	117	46.6		11 (9.4)	106 (90.6)	<0.001
<80%	84	33.5		6 (7.1)	78 (92.9)	<0.001
Case definition and diagnosis						
Positive diagnosis (*n* = 219)						0.327
Rapid lateral flow test positive to IgG	12	5.5		4 (33.3)	8 (66.7)	
Rapid lateral flow test positive to IgM/IgG	178	81.3		55 (30.9)	123 (69.1)	
Rapid lateral flow test positive to IgM	22	10.1		5 (22.7)	17 (77.3)	
RT-PCR	7	3.2		0 (0)	7 (100)	
Case definition						0.102
Suspicious	43	16.4		18 (41.9)	25 (58.1)	
Confirmed	219	83.6		64 (29.2)	155 (70.8)	
Baseline laboratory markers						
Hemoglobin (g/dL) (*n* = 207)			13.3 (12.1-14.1)	13.7 (12.1-14.5)	13.3 (12.1-14.0)	0.159
Leukocytes (cells/mm^3^) (*n* = 262)			11.8 (8.5-15.9)	8.7 (7.0-12.7)	12.8 (9.5-17.6)	<0.001
Leukocytosis (≥10.000 cells/mm^3^)	167	63.7		34 (20.4)	133 (79.6)	<0.001
Neutrophils, cells/mm^3^ (*n* = 262)			10.5 (7.3-14.5)	7.4 (5.2-11.7)	11.7 (8.7-16.4)	<0.001
Lymphocytes (cells/mm^3^) (*n* = 262)			0.8 (0.5-1.3)	0.9 (0.6-1.4)	0.8 (0.5-1.1)	0.129
Lymphopenia (<0.8 cells/mm^3^)	120	45.8		32 (26.7)	88 (73.3)	0.137
Platelets (cells/mm^3^) (*n* = 262)			262 (196-357)	276 (199-365)	259 (194-356)	0.487
Thrombocytopenia (<150.000 cells/mm^3^)	35	13.4		10 (28.6)	25 (71.4)	0.709
Creatinine (mg/dL) (*n* = 212)			0.78 (0.59-0.97)	0.75 (0.58-0.88)	0.79 (0.60-1.03)	0.159
Time						
Length of hospital stay in days			5 (3-9)	9 (5-14)	4 (2-7)	<0.001
Medical requirements						
Admitted to ICU	7	2.7		0 (0)	7 (100)	0.07
High flow oxygen requirement (FiO_2_ ≥ 0.36)	199	80.6		23 (11.6)	176 (88.4)	<0.001
ICU requirement (FiO_2_ ≥ 0.80)	191	72.9		18 (9.4)	173 (90.6)	<0.001

Data expressed as mean ± standard deviation, median (interquartile range) or number (percentage). ^1^SD: standard deviation; NLR: neutrophil-to-lymphocyte ratio; PLR: platelet-to-lymphocyte ratio; RT-PCR: reverse transcription polymerase chain reaction; ICU: intensive care unit.

**Table 2:** Descriptive and bivariate analysis of the treatment received according to in-hospital death in older adults hospitalized for COVID-19 (*n* = 262).

Variables	*n*	%	In-hospital death		*P* value
			Survivor	Nonsurvivor	
			*n* = 82	n = 180	
			31.30%	68.70%	
Received antibiotic therapy					0.939
No	3	1.1	1 (33.3)	2 (66.7)	
Yes	259	98.9	81 (31.3)	178 (68.7)	
Types of antibiotic therapy					0.768
Azithromycin+cephalosporins	205	79.1	67 (32.7)	138 (67.3)	
Azithromycin	21	8.1	4 (19.1)	17 (80.9)	
Cephalosporins	13	5	5 (38.5)	8 (61.5)	
Azithromycin+carbapenems	16	6.2	4 (25.0)	12 (75.0)	
Carbapenems	3	1.2	1 (33.3)	2 (66.7)	
Azithromycin+others	1	0.4	0 (0)	1 (100)	
Received corticosteroids					0.445
No	81	30.9	28 (34.6)	53 (65.4)	
Yes	181	69.1	54 (29.8)	127 (70.2)	
Type of corticosteroids					<0.001
Methylprednisolone	110	60.8	26 (23.6)	84 (76.4)	
Dexamethasone	56	30.9	26 (46.4)	30 (53.6)	
Hydrocortisone	13	7.2	0 (0)	13 (100)	
Prednisone	2	1.1	2 (100)	0 (0)	
Received hydroxychloroquine					0.978
No	70	26.7	22 (31.4)	48 (68.6)	
Yes	192	73.3	60 (31.3)	132 (68.7)	
Received ivermectin					0.042
No	191	72.9	53 (27.7)	138 (72.3)	
Yes	71	27.1	29 (40.9)	42 (59.1)	
Received enoxaparin					0.045
No	54	20.6	23 (42.6)	31 (57.4)	
Yes	208	79.4	59 (28.4)	149 (71.6)	
Received lopinavir/ritonavir					0.786
No	205	78.2	65 (31.7)	140 (68.3)	
Yes	57	21.8	17 (29.8)	40 (70.2)	

Data expressed as number (percentage).

**Table 3:** Cox regression analysis to assess the role of tertiles of the neutrophil-to-lymphocyte ratio and the platelet-to-lymphocyte count ratio as risk factors for mortality in adults hospitalized for COVID-19.

Variables	Crude model (*n* = 262)		Adjusted model^1^	
	cHR (95% CI)	*P* value	aHR (95% CI)	*P* value
Model 1: NLR		<0.001		0.017
Low tertile	Reference		Reference	
Intermediate tertile	1.89 (1.29-2.78)		1.35 (0.90-2.04)	
High tertile	2.37 (1.62-3.45)		2.12 (1.43-3.14)	
Model 2: PLR		0.004		0.004
Low tertile	Reference		Reference	
Intermediate tertile	1.47 (1.01-2.14)		1.29 (0.86-1.94)	
High tertile	1.84 (1.28-2.65)		1.90 (1.30-2.79)	
Model 3: logarithm of NLR	1.65 (1.37-1.98)	<0.001	1.56 (1.30-1.88)	<0.001
Model 4: logarithm of PLR	1.37 (1.15-1.53)	<0.001	1.42 (1.19-1.71)	<0.001

HR: hazard ratio; cHR: crude hazard ratio; aHR: adjusted hazard ratio; 95% CI: 95% confidence interval; NLR: neutrophil-to-lymphocyte ratio; PLR: platelet-to-lymphocyte ratio. ^1^Models adjusted for: age (nonlinear form), sex, comorbidities, oxygen saturation (nonlinear form), and treatment with dexamethasone.

**Table 4:** Optimal cut-off points to predict 60-day mortality for NLR and PLR based on Youden's index and maximally selected rank statistics criteria.

Method of estimate cut-off point	Cut-off point	Sensitivity % (95% CI)	Specificity % (95% CI)
NLR			
Youden's index	8.6	79.4 (73.5 to 85.4)	55.4 (42.3 to 68.4)
Maximally selected rank statistics	6.23	90.6 (86.3 to 94.8)	39.3 (26.5 to 52.1)
PLR			
Youden's index	34.2	62.8 (55.7 to 69.9)	69.6 (57.6 to 81.7)
Maximally selected rank statistics	34.2	62.8 (55.7 to 69.9)	69.6 (57.6 to 81.7)

95% CI: 95% confidence interval; NLR: neutrophil-to-lymphocyte ratio; PLR: platelet-to-lymphocyte ratio.

## Data Availability

The datasets analyzed in this research article are not publicly available. Any request can be addressed to diego.urrunaga.pastor1@gmail.com.

## Supplementary Materials

Supplementary Materials Figure S1: univariable distribution of NLR an PLR in the population study. Figure S2: distribution of NLR an PLR according to death status at the last follow-up. Figure S3: adjusted relationship between NLR and PLR with mortality. Reference value for NLR was 1.875 and for PLR was 3.333. Figure S4: crude relationship between NLR and PLR with mortality. Reference value for NLR was 1.875 and for PLR was 3.333. Figure S5: adjusted relationship between age and oxygen saturation with mortality in model 3. Reference value for age was 60 and for oxygen saturation was 98%. Figure S6: adjusted relationship between age and oxygen saturation with mortality in model 4. Reference value for age was 60 and for oxygen saturation was 98%. Figure S7: area under the curve ROC and 95% confidence bands of (A) NLR and (B) PLR to predict mortality at 15, 30, and 60 days. Figure S8: determination of cut-off point for NLR using maximally selected rank statistics. Figure S9: determination of cut-off point for PLR using maximally selected rank statistics. Figure S10: K-M survival curves for NLR and PLR categorized according to maximal selected rank statistics.
