# Supplementary material for: The Neutrophil-to-Lymphocyte Ratio and the Platelet-to-Lymphocyte Ratio as Predictors of Mortality in Older Adults Hospitalized with COVID-19 in Peru
Source: Dis Markers. 2022 Aug 3;2022:2497202. doi: 10.1155/2022/2497202 (PMC9346540; doi:10.1155/2022/2497202)
Supplement: Supplementary Materials — Figure S1: univariable distribution of NLR an PLR in the population study. Figure S2: distribution of NLR an PLR according to death status at the last follow-up. Figure S3: adjusted relationship between NLR and PLR with mortality. Reference value for NLR was 1.875 and for PLR was 3.333. Figure S4: crude relationship between NLR and PLR with mortality. Reference value for NLR was 1.875 and for PLR was 3.333. Figure S5: adjusted relationship between age and oxygen saturation with mortality in model 3. Reference value for age was 60 and for oxygen saturation was 98%. Figure S6: adjusted relationship between age and oxygen saturation with mortality in model 4. Reference value for age was 60 and for oxygen saturation was 98%. Figure S7: area under the curve ROC and 95% confidence bands of (A) NLR and (B) PLR to predict mortality at 15, 30, and 60 days. Figure S8: determination of cut-off point for NLR using maximally selected rank statistics. Figure S9: determination of cut-off point for PLR using maximally selected rank statistics. Figure S10: K-M survival curves for NLR and PLR categorized according to maximal selected rank statistics. [file 2497202.f1.docx]

**Supplementary material**

Figure S1. Univariable distribution of NLR an PLR in the population study.

**
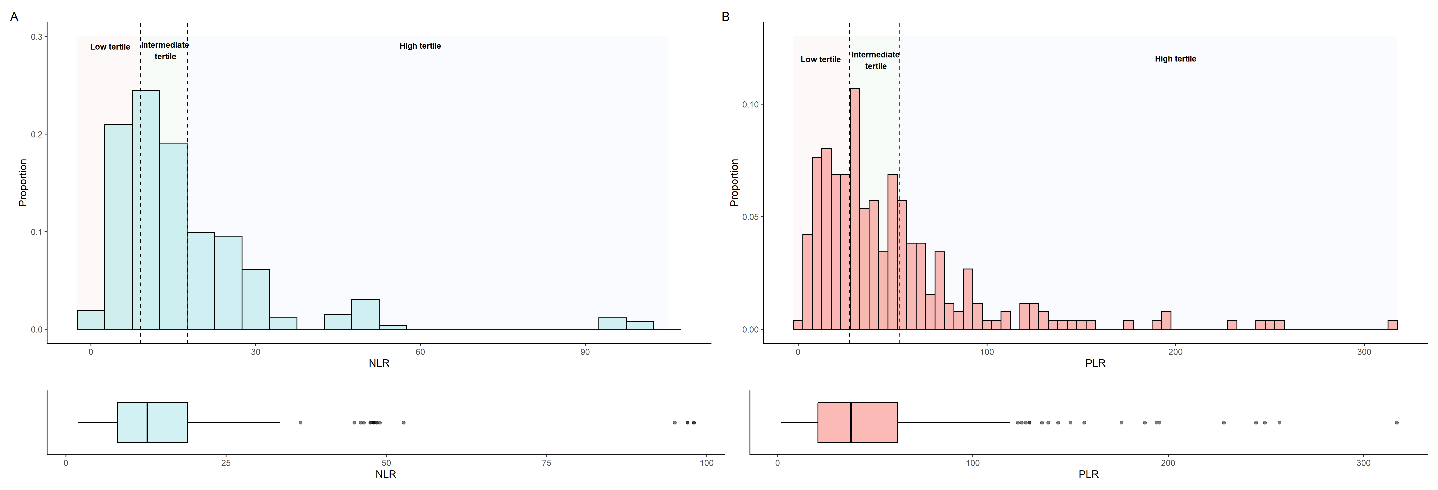
**

Figure S2. Distribution of NLR an PLR according to death status at the last follow-up.

**
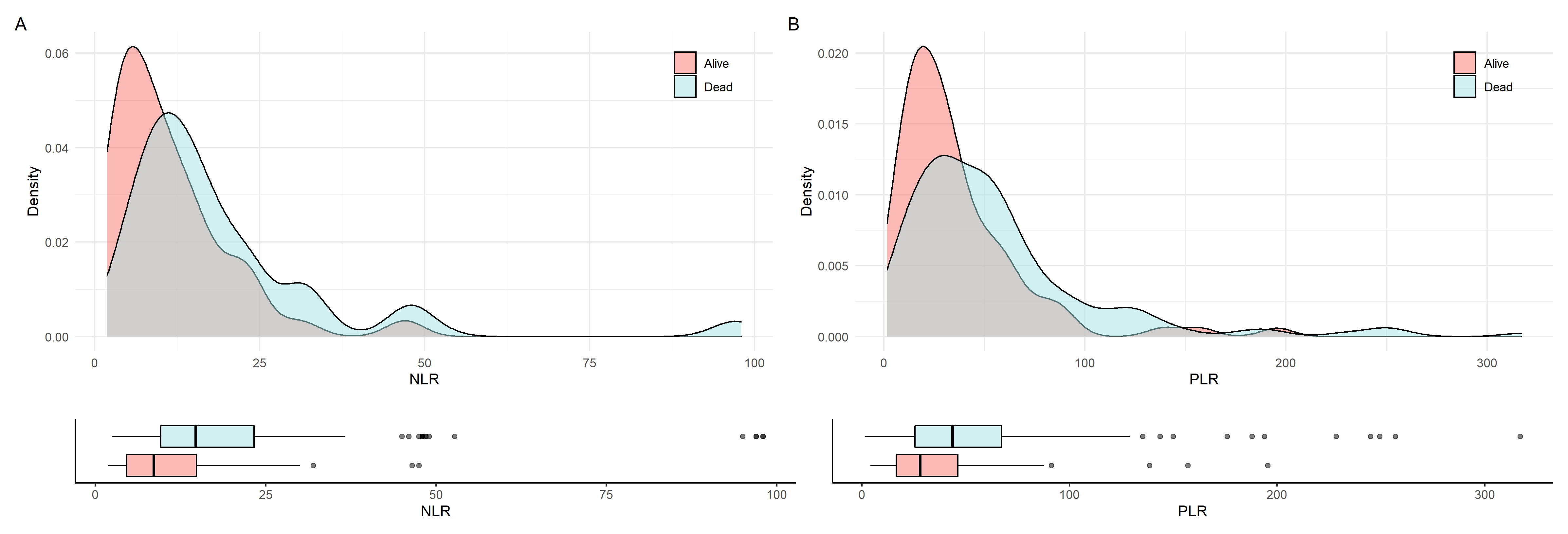
**

Figure S3. Adjusted relationship between NLR and PLR with mortality. Reference value for NLR was 1.875 and for PLR was 3.333.
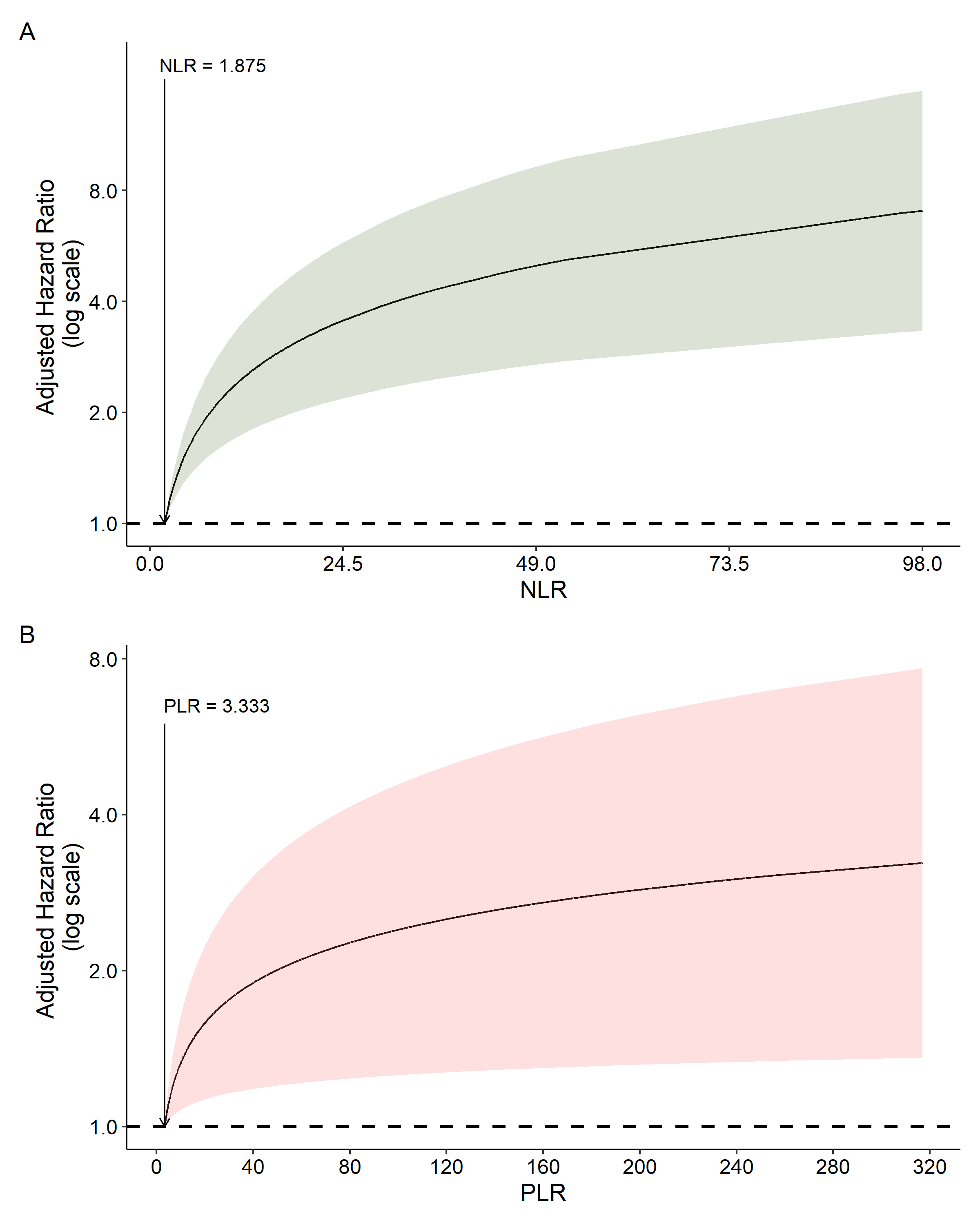


Figure S4. Crude relationship between NLR and PLR with mortality. Reference value for NLR was 1.875 and for PLR was 3.333.


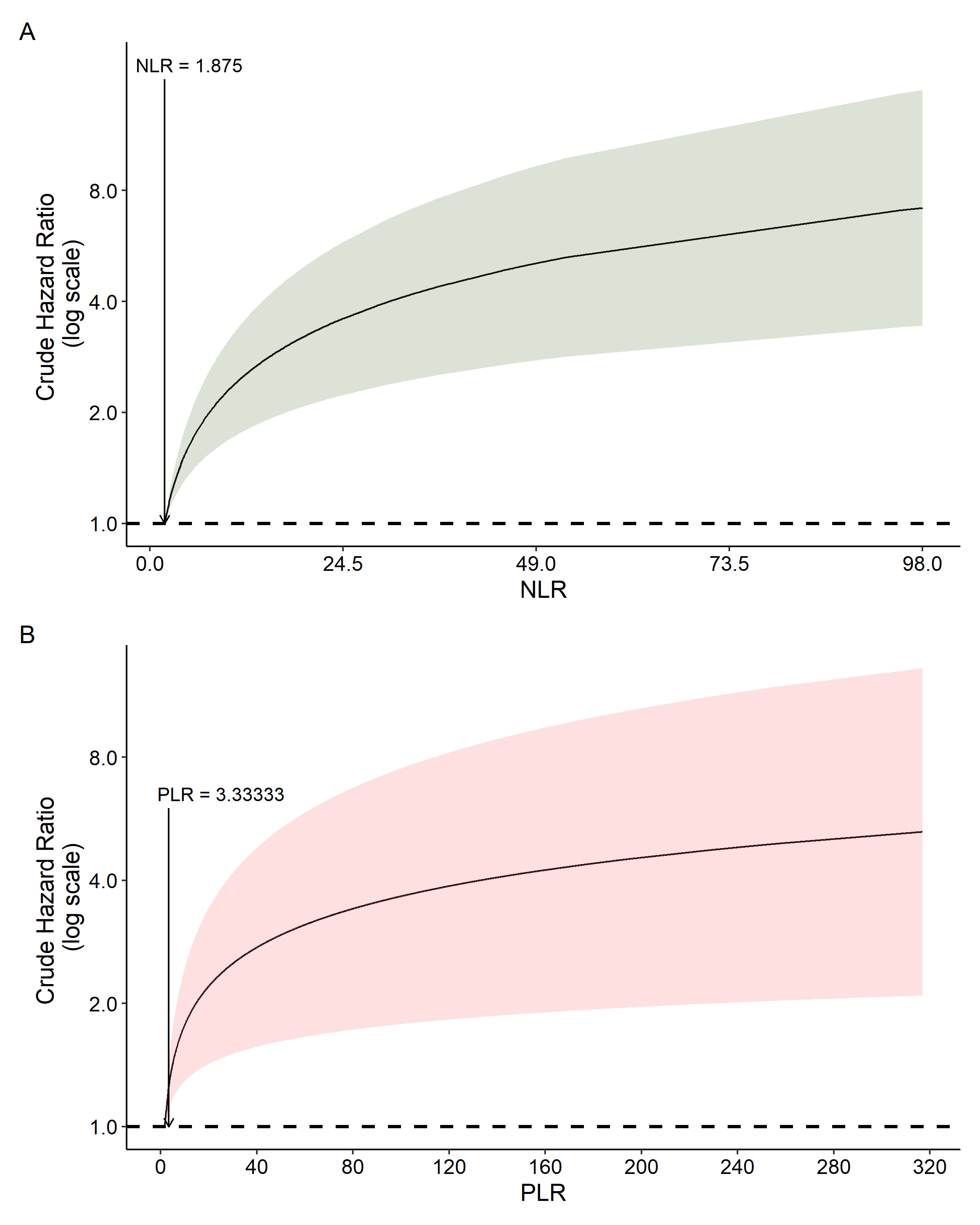


Figure S5. Adjusted relationship between age and oxygen saturation with mortality in model 3. Reference value for age was 60 and for oxygen saturation was 98%.


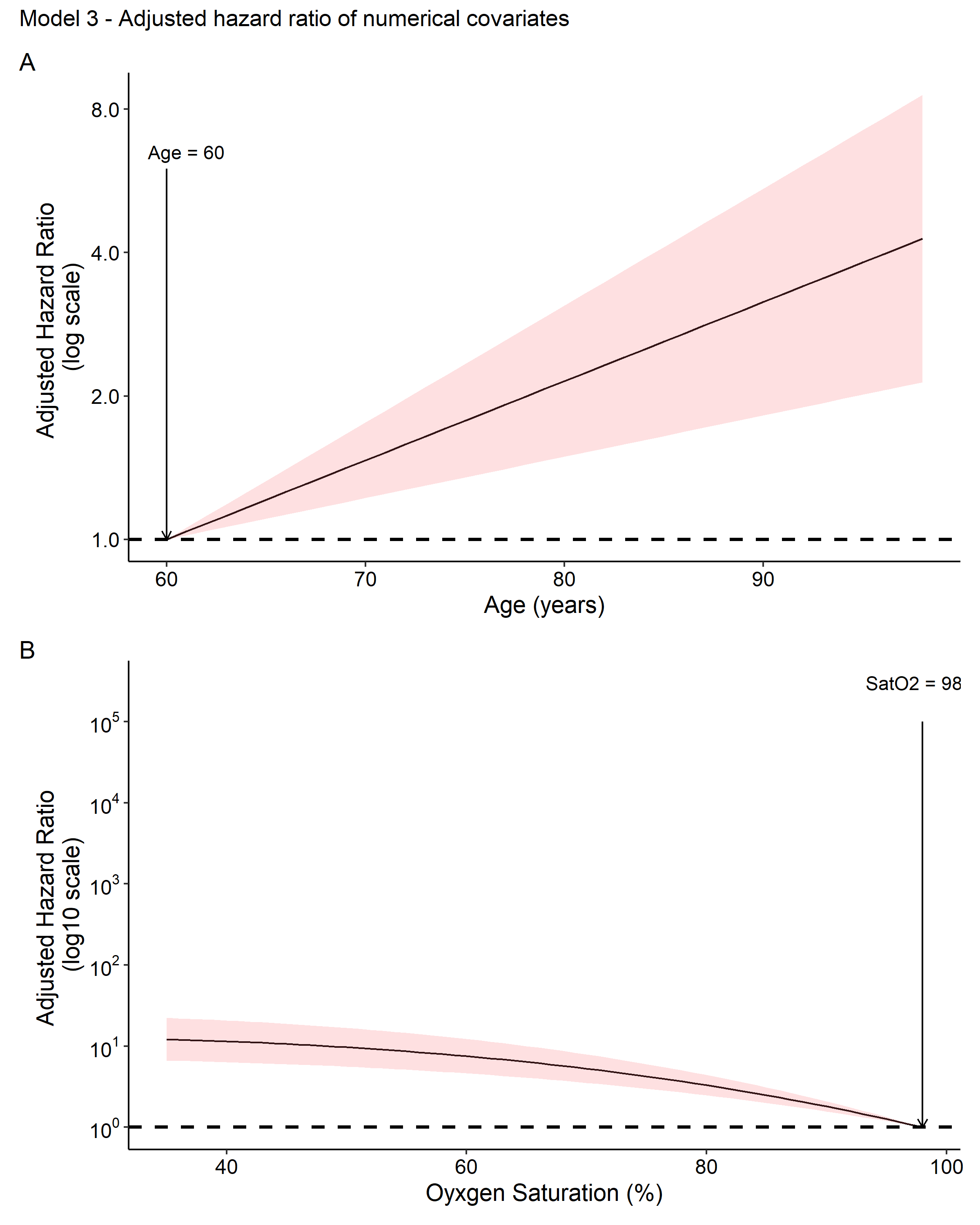


Figure S6. Adjusted relationship between age and oxygen saturation with mortality in model 4. Reference value for age was 60 and for oxygen saturation was 98%.


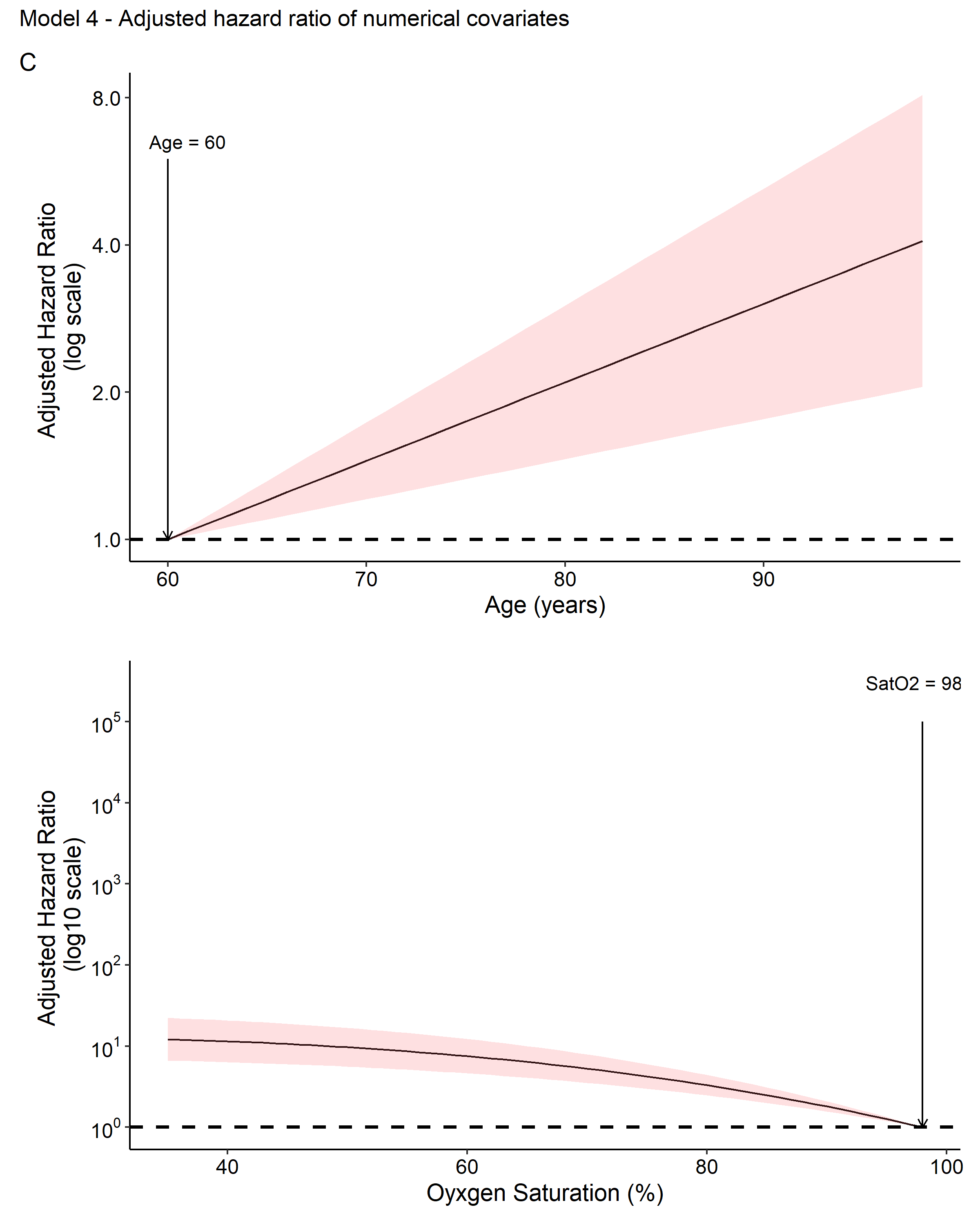


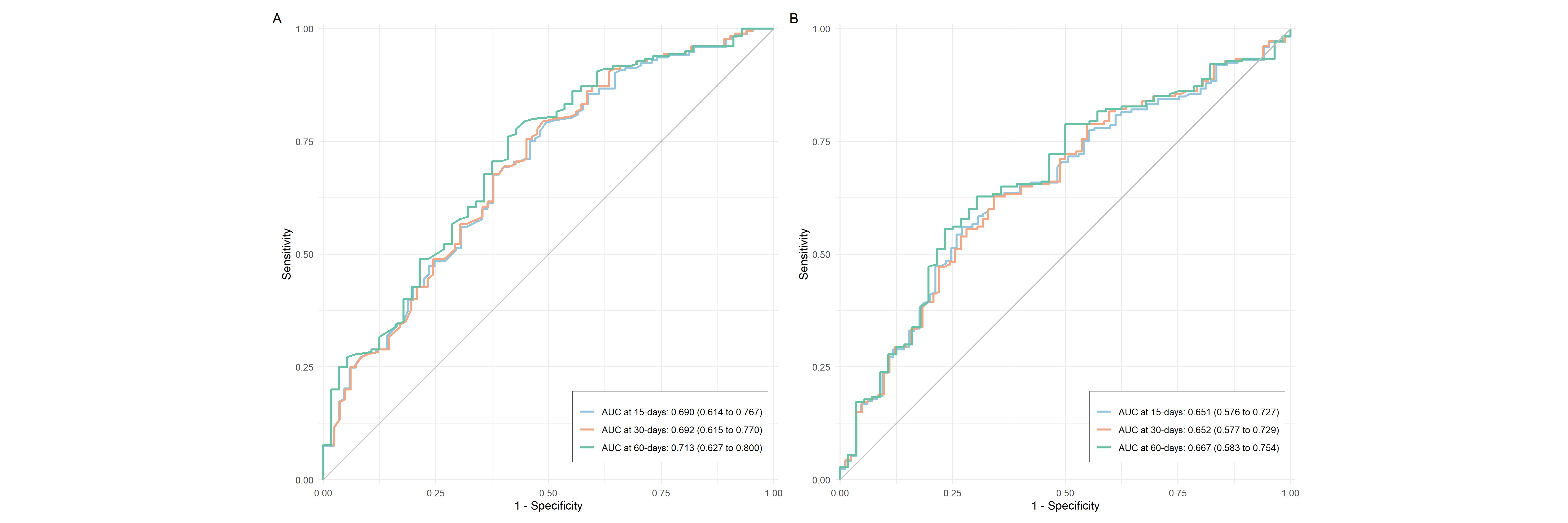
Figure S7. Area under the curve ROC and 95% confidence bands of (A) NLR and (B) PLR to predict mortality at 15, 30 and 60 days.

Figure S8. Determination of cut-off point for NLR using maximally selected rank statistics.


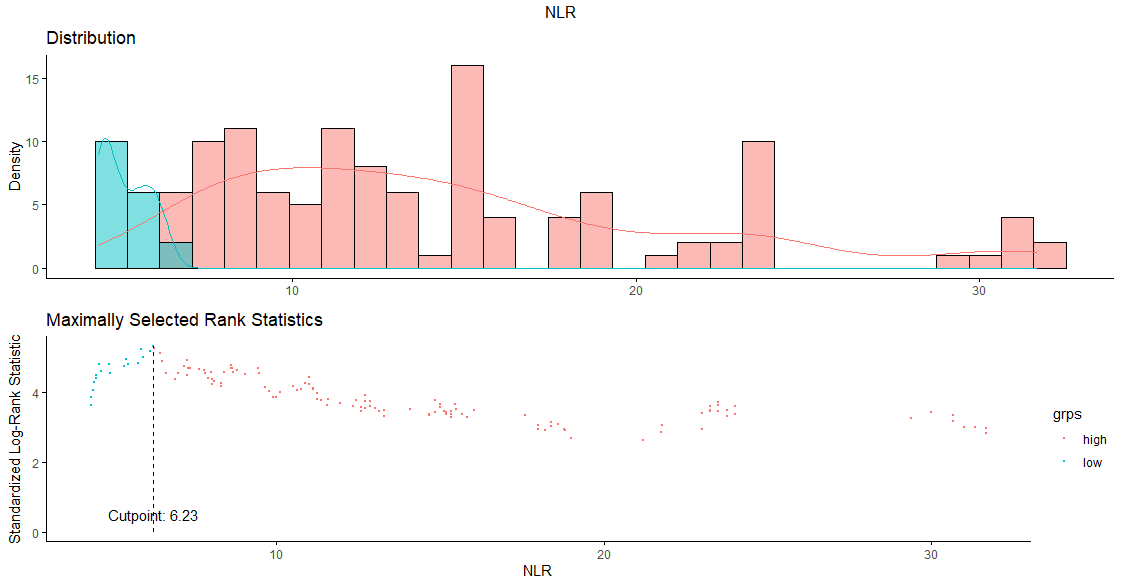


Figure S9. Determination of cut-off point for PLR using maximally selected rank statistics


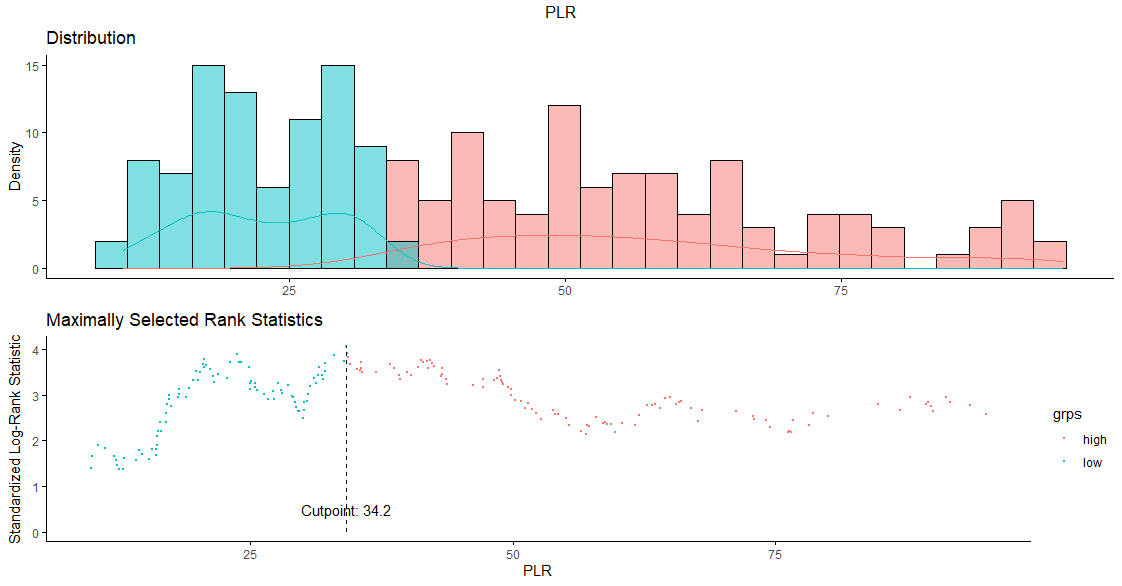


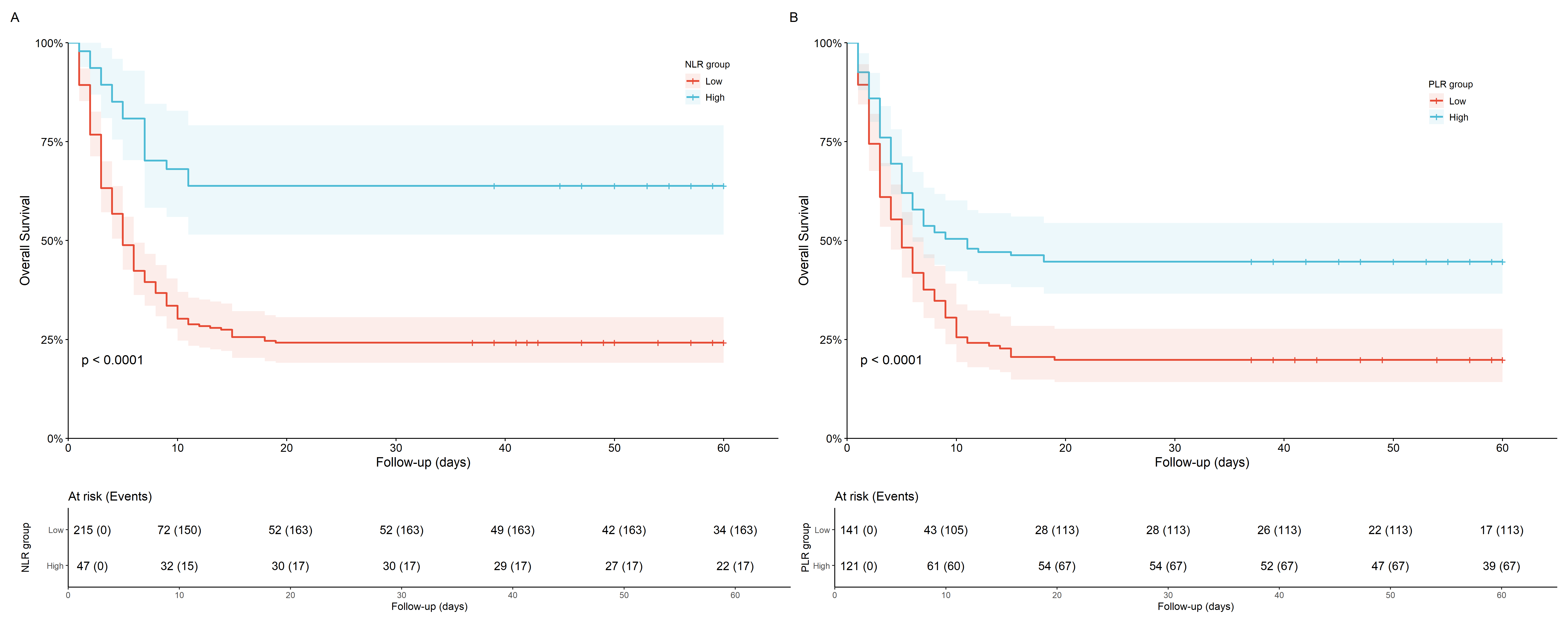
Figure S10. K-M survival curves for NLR and PLR categorized according to maximal selected rank statistics.
